# Oncological and surgical outcomes of radical surgery in elderly colorectal cancer patients with intestinal obstruction

**DOI:** 10.3389/fsurg.2023.1251461

**Published:** 2023-08-22

**Authors:** Qingbiao Ma, Hongyu Li, Yujuan Jiang, Yingfei Wang, Jianwei Liang

**Affiliations:** ^1^ Department of General Surgery, Liaoyang Central Hospital, Liaoyang, China; ^2^ Department of Colorectal Surgery, National Cancer Center/National Clinical Research Center for Cancer/Cancer Hospital, Chinese Academy of Medical Sciences and Peking Union Medical College, Beijing, China

**Keywords:** colorectal cancer, elderly, radical surgery, palliative surgery, safety, prognoses

## Abstract

**Background:**

The treatment strategy for elderly colorectal cancer patients with intestinal obstruction remains controversial. The choice of reasonable treatment and surgical method directly affects perioperative safety and prognosis. This study investigated the safety and long-term efficacy of radical surgery in elderly colorectal cancer patients over 80 years old with intestinal obstruction.

**Methods:**

The clinicopathological data of elderly patients over 80 years old with intestinal obstruction who underwent colorectal cancer surgery from January 2012 to December 2021 were retrospectively collected and analysed. Patients were assigned to a radical group and a palliative group according to the surgical method. Propensity score matching (PSM) was performed to match patients in the radical group 1:1 with those in the palliative group. The perioperative-related indexes and prognosis were compared between the two groups.

**Results:**

A total of 187 patients were enrolled in this study. After PSM, 58 matched pairs were selected, and the radical and palliative groups were well balanced in terms of the clinical and surgical characteristics (*P *> 0.05). The proportion of patients transferred to the ICU after surgery in the radical group was significantly higher than that in the palliative group (17.2% vs. 5.2%, *P *= 0.039). In terms of postoperative complications, the incidence of grade 1–5 complications in the radical group was significantly higher than that in the palliative group (37.9% vs. 15.5%, *P *= 0.006); however, there was no significant difference in the incidence of grade 3–5 complications between the two groups (6.9% vs. 1.7%, *P *= 0.364). In addition, the complications were subclassified, and it was found that the incidence of gastrointestinal disorders (20.7% vs. 6.9%, *P *= 0.031) after surgery was significantly higher in the radical group. The 3-year OS rates were 55.2% and 22.6% in the radical and palliative groups, respectively (*P *< 0.001). Multivariate analysis revealed that radical surgery was an independent prognostic factor for OS (HR: 4.32; 95% CI, 1.93–12.45; *P *< 0.001).

**Conclusion:**

Although elderly colorectal cancer patients over 80 years of age with intestinal obstruction are more likely to be admitted to the ICU and develop more postoperative complications after radical surgery, long-term survival benefits can be achieved.

## 1. Introduction

Colorectal cancer is the second leading cause of cancer death in the United States; approximately 153,020 individuals will be diagnosed with colorectal cancer, and 52,550 will die from the disease in 2023 (1–3). The incidence of colorectal cancer is positively correlated with age. With the aging of the population, the proportion of elderly patients with colorectal cancer continues to increase (4–6). Elderly colorectal cancer patients often experience an insidious onset of disease, and the disease is often in the advanced stages at initial diagnosis. Intestinal obstruction, as a common first symptom in advanced colorectal patients, is likely to lead to systemic disorders such as malnutrition and water-electrolyte imbalance in elderly patients, potentially increasing the incidence of perioperative complications and mortality. Radical surgery is the main potentially curative treatment for colorectal cancer patients. However, radical surgery involves tumor resection and gastrointestinal reconstruction, and once postoperative complications such as anastomotic leakage, pelvic infection, and cerebrovascular disorders occur, they will significantly affect the quality of life and prognosis of elderly colorectal cancer patients with intestinal obstruction and even cause death (7, 8). Therefore, for elderly colorectal cancer patients over 80 years old with intestinal obstruction, surgeons and patients' families often choose more conservative treatment strategies for the purpose of reducing symptoms and improving quality of life for various reasons. It is well known that the choice of reasonable treatment and surgical method directly affects the perioperative safety and prognosis of patients with colorectal cancer (9–11). Therefore, this study aimed to investigate and compare the safety and long-term survival benefits of radical surgery and palliative surgery in elderly colorectal cancer patients over 80 years of age with intestinal obstruction.

## 2. Patients and methods

### 2.1. Patients

In this study, we retrospectively analyzed the clinicopathological data of curable elderly patients who underwent colorectal surgery at the Cancer Hospital of the Chinese Academy of Medical Sciences from January 2012 to December 2021. The inclusion criteria were as follows: (1) age ≥80 years; (2) preoperative intestinal obstruction; (3) pathological diagnosis of adenocarcinoma; and (4) American Society of Anesthesiologists (ASA) scores 1–3 or ECOG score 0–2. The exclusion criteria were as follows: (1) distant metastasis; (2) emergency surgery; (3) adjuvant therapy; (4) preoperative therapy; (5) other malignancies; and (6) no chance of radical resection. Intestinal obstruction is defined as a colonoscopy showing tumor growth beyond 1/2 diameter of the lumen, accompanied by proximal intestinal dilatation with gas and fluid accumulation. All patients signed informed consent before surgery, and the design and conduct of this study were approved by the Ethics Committee of the institution. The study conformed to the ethical standards of the World Medical Association Declaration of Helsinki, and all methods were carried out in accordance with relevant guidelines and regulations.

### 2.2. Preoperative diagnosis and treatment

All patients were diagnosed and treated in accordance with National Comprehensive Cancer Network (NCCN) guidelines. All patients were required to undergo laboratory examination, colonoscopy, chest, abdomen and pelvis CT before surgery to identify tumor conditions and exclude distant metastasis. Electrocardiogram, echocardiogram, pulmonary function, and, if necessary, 24-hour ambulatory electrocardiogram or coronary angiography were performed to determine the patient's cardiopulmonary function. All patients were discussed in multidisciplinary treatment meetings that included surgical oncologists, medical oncologists and anesthetists. All enrolled patients were eligible for radical resection, the risks and benefits of surgery were explained to the patients and their families, and the treatment plan was decided by the patients. According to whether the tumor was completely radically removed, all patients were classified into a radical group and a palliative group. Palliative surgeries include ostomy, intestinal short-circuiting, and intestinal stent placement. Open or laparoscopic surgery is selected according to the patient's own wishes and the surgeon's evaluation. Before 2015, open surgery was the mainstay, and then laparoscopic surgery continued to develop and became the main surgical approach. Mechanical anastomosis was used in the reconstruction of the digestive tract. Before surgery, hypoproteinaemia and anemia were required to be improved, serum albumin was required to have increased to more than 30 g/L, and hemoglobin was required to be increased to more than 90 g/L.

In this study, baseline data were collected based on electronic records and included age, sex, body mass index (BMI), preoperative hemoglobin (HGB) level, preoperative albumin level, ASA score, comorbidities, previous abdominal surgery, tumor location, clinical TNM stage, tumor differentiation, date of surgery, and surgical approach. In addition, data regarding the surgical outcomes were also collected, including the operative time, estimated blood loss, ICU admission, postoperative complications, mortality, time to first flatus and postoperative hospital stay. Postoperative complications were graded according to the Clavien‒Dindo surgical grading system, and grade 3–5 complications were defined as severe complications (12). According to the origin of complications, complications were classified as cardiac disorders, respiratory disorders, gastrointestinal disorders, renal and urinary disorders, and other disorders.

### 2.3. Survival analysis

All patients had regular outpatient or telephone follow-up after surgery. Follow-up was conducted every 3 months for the first 2 years and every 6 months after 2 years. The components of outpatient follow-up examination included physical examination, tumor markers, colonoscopy, chest, abdomen and pelvis CT. The end point of this study was 5-year overall survival (OS). OS was defined as the time elapsed from the date of tumor diagnosis to death from any cause.

### 2.4. Statistical analysis

Statistical analysis was performed by SPSS Statistics 25.0 for Windows (IBM Corp, Armonk, NY, USA) in this study. To reduce the imbalance between the two groups, propensity score matching (PSM) was performed to match patients in the radical group 1:1 with those in the palliative group (caliper = 0.2), and the covariates included age, sex, BMI, preoperative HGB level, preoperative albumin level, ASA score, comorbidity, previous abdominal surgery, tumor location, clinical TNM stage, tumor differentiation, date of surgery, and surgical approach.

Continuous variables are expressed as the mean ± standard deviation, and *t-*tests were used for comparisons between groups. Categorical variables are expressed as numbers (%), and comparisons were made between groups using either the *χ*^2^ test or Fisher's exact test. The Kaplan‒Meier method was used for survival analysis, and the log-rank method was used for comparisons between groups. Variables with significant differences were included in the Cox proportional hazard regression model for multivariate analysis. A *P* value less than 0.05 was considered statistically significant.

## 3. Results

### 3.1. Baseline data

A total of 187 patients were identified from the electronic database and were eligible for inclusion. Among them, 114 and 73 were assigned to the radical group and palliative group, respectively. Using the PSM method, 58 matched pairs were selected.

Baseline data before and after matching between the groups are presented in Table 1. The proportion of patients with preoperative comorbidities in the palliative group was significantly higher than that in the radical group (65.8% vs. 44.7%, *P *= 0.005), resulting in a significantly higher proportion of patients with ASA grade 3 in the palliative group (60.3% vs. 40.4%, *P *= 0.008). In addition, the proportion of patients in the palliative group who underwent surgery before January 1, 2017, was significantly higher than that in the radical group. After PSM, the radical and palliative groups were well balanced in terms of the abovementioned variables (*P *> 0.05).

**Table 1:** Clinical and surgical characteristics before and after matching between radical and palliative groups.

Variables	Total cohort			Matched cohort		
	Radical group (*n* = 114)	Palliative group (*n* = 73)	*P*	Radical group (*n* = 58)	Palliative group (*n* = 58)	*P*
Age (years, mean ± SD)	81.4 ± 2.4	82.3 ± 2.1	0.482	81.6 ± 2.2	82.2 ± 2.0	0.436
Gender (%)			0.563			0.444
Male	67 (58.8)	46 (63.0)		34 (58.6)	38 (65.5)	
Female	47 (41.2)	27 (37.0)		24 (41.4)	20 (34.5)	
Body mass index (kg/m^2^, mean ± SD)	23.5 ± 2.5	23.0 ± 2.3	0.490	23.3 ± 2.5	23.0 ± 2.3	0.628
Preoperative HGB level (g/L, mean ± SD)	121.3 ± 22.3	114.8 ± 20.2	0.102	118.5 ± 21.1	115.2 ± 20.5	0.556
Preoperative albumin level (g/L, mean ± SD)	37.4 ± 4.1	35.0 ± 3.9	0.140	36.2 ± 3.7	35.6 ± 3.9	0.331
ASA classification (%)			0.008			0.353
I–II	68 (59.6)	29 (39.7)		31 (53.4)	26 (44.8)	
III	46 (40.4)	44 (60.3)		27 (46.6)	32 (55.2)	
Comorbidity (%)	51 (44.7)	48 (65.8)	0.005	32 (55.2)	37 (63.8)	0.344
Previous abdominal surgery (%)	20 (17.5)	11 (15.1)	0.657	8 (13.8)	8 (13.8)	1.000
Tumor location (%)			0.334			0.836
Right colon	62 (54.4)	39 (53.4)		29 (50.0)	31 (53.4)	
Left colon	46 (40.4)	26 (35.6)		24 (41.4)	21 (36.2)	
Rectum	6 (5.2)	8 (11.0)		5 (8.6)	6 (10.3)	
Clinical TNM stage (%)			0.155			0.576
II	59 (51.8)	30 (41.0)		28 (48.3)	25 (43.1)	
III	55 (48.2)	43 (59.0)		30 (51.7)	33 (56.9)	
Tumor differentiation			0.696			0.461
Well	10 (8.8)	4 (5.5)		8 (13.8)	4 (6.9)	
Moderate	81 (71.1)	53 (72.6)		39 (67.2)	41 (70.7)	
Poor	23 (20.1)	16 (21.9)		11 (19.0)	13 (22.4)	
Date of Surgery (%)			0.048			0.331
Before January 1 2017	31 (27.2)	30 (41.1)		18 (31.0)	23 (39.7)	
After January 1 2017	83 (72.8)	43 (58.9)		40 (69.0)	35 (60.3)	
Surgical approach (%)			0.062			0.263
Laparoscopic	72 (63.2)	36 (49.3)		35 (60.3)	29 (50.0)	
Open	42 (36.8)	37 (50.7)		23 (39.7)	29 (50.0)	

### 3.2. Short-term outcomes

The short-term outcomes, including the surgical data, postoperative complications, and postoperative recovery, in the matched cohorts are summarized in Table 2. The average operation time (158.8 vs. 87.3 min, *P *< 0.001) in the radical group was significantly longer than that in the palliative group. In addition, the average estimated blood loss in the radical group was higher (70.7 vs. 32.3 ml, *P *= 0.084), but no significant difference was achieved. In addition, the proportion of patients transferred to the ICU after surgery in the radical group was significantly higher than that in the palliative group (17.2% vs. 5.2%, *P *= 0.039). In terms of postoperative complications, the incidence of grade 1–5 complications in the radical group was significantly higher than that in the palliative group (37.9% vs. 15.5%, *P *= 0.006); however, there was no significant difference in the incidence of grade 3–5 complications between the two groups (6.9% vs. 1.7%, *P *= 0.364). In addition, the complications were subclassified, and it was found that the incidence of gastrointestinal disorders (20.7% vs. 6.9%, *P *= 0.031) after surgery was significantly higher in the radical group. The postoperative hospital stay in the radical group was significantly longer than that in the palliative group (9.5 vs. 5.3 days, *P *< 0.001). There were no deaths within 30 days during the perioperative period.

**Table 2:** Perioperative data of patients in the radical and palliative groups.

Characteristics	Radical group (*n* = 58)	Palliative group (*n* = 58)	*P*
Operative time (min, mean ± SD)	158.8 ± 60.5	87.3 ± 40.5	<0.001
Estimated blood loss (ml, mean ± SD)	70.7 ± 30.1	32.3 ± 10.4	0.084
ICU admission	10 (17.2)	3 (5.2)	0.039
Postoperative complications (grade-1–5)	22 (37.9)	9 (15.5)	0.006
Cardiac disorders	4 (6.9)	1 (1.7)	0.364
Arrhythmia	3 (5.2)	1 (1.7)	0.618
Cardiac failure	2 (3.4)	0 (0)	0.496
Acute coronary syndrome	1 (1.7)	0 (0)	1.000
Pulmonary embolism	0 (0)	1 (1.7)	1.000
Respiratory disorder	4 (6.9)	2 (3.4)	0.679
Pneumonia	3 (5.2)	1 (1.7)	0.618
Pleural effusion	1 (1.7)	1 (1.7)	1.000
Atelectasis	1 (1.7)	1 (1.7)	1.000
Gastrointestinal disorders	12 (20.7)	4 (6.9)	0.031
Anastomotic leakage	4 (6.9)	0 (0)	0.119
Ileus	6 (10.3)	3 (5.2)	0.490
GastrointestinaI haemorrhage	1 (1.7)	0 (0)	1.000
Gastroparesis	4 (6.9)	2 (3.4)	0.679
Renal and urinary disorders	5 (8.6)	1 (1.7)	0.206
Urinary infection	0 (0)	1 (1.7)	1.000
Renal failure	1 (1.7)	0 (0)	1.000
Urinary retention	4 (6.9)	1 (1.7)	0.364
Other disorders	8 (13.8)	4 (6.9)	0.223
Abdominal abscess	2 (3.4)	0 (0)	0.496
Intra-abdominal haemorrhage	0 (0)	1 (1.7)	1.000
Wound infection	4 (6.9)	2 (3.4)	0.679
Cerebral infarction	1 (1.7)	0 (0)	1.000
Delirium	3 (5.2)	2 (3.4)	1.000
Postoperative complications (grade 3–5)	4 (6.9)	1 (1.7)	0.364
Mortality with in 30 days (%)	0 (0)	0 (0)	–
Time to first flatus (days, mean ± SD)	3.2 ± 1.6	2.6 ± 1.3	0.143
Postoperative hospital stay (days, mean ± SD)	9.5 ± 2.9	5.3 ± 3.2	<0.001

### 3.3. Survival analysis

**Figure 1:**
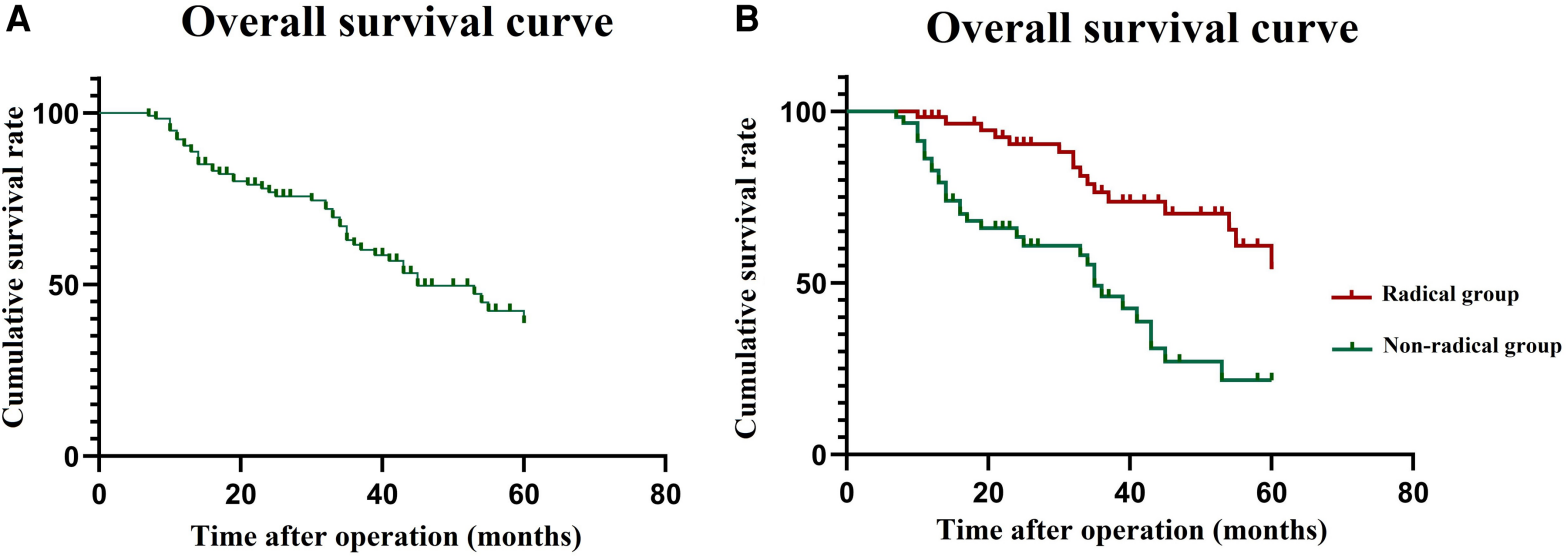
Survival curve in the matched cohort. (**A**) overall survival curve for 116 patients; (**B**) overall survival curve of the radical group and palliative group.

In the matched cohort, during follow-up, three patients (two in the radical group and one in the palliative group) were lost to follow-up. The follow-up period was 13–117 months, and the median follow-up time was 58 months. The median follow-up periods for the radical group and palliative groups were 61 months and 56 months, respectively. The 5-year OS rate for patients in the matched cohort was 38.7% (Figure 1A). The 3-year OS rates were 55.2% and 22.6% in the radical and palliative groups, respectively (*P *< 0.001) (Figure 1B). The univariate and multivariate analyses of the prognostic factors influencing OS are presented in Table 3. In univariate analysis, comorbidity, clinical TNM stage and radical surgery significantly affected OS (*P *< 0.05). Multivariate analysis revealed that radical surgery was an independent prognostic factor for OS (HR: 4.32; 95% CI, 1.93–12.45; *P *< 0.001).

**Table 3:** The univariate and multivariate analyses of the prognostic factors influencing OS.

Variables	Overall survival			
	Univariate analysis		Multivariate analysis	
	HR (95% CI)	*P*	HR (95% CI)	*P*
Gender: male/female	1.34 (0.65–2.79)	0.433		
Age at operation	1.03 (0.89–1.20)	0.410		
Preoperative HGB level	0.99 (0.97–1.01)	0.492		
Preoperative albumin level	0.95 (0.89–1.04)	0.483		
ASA classification (III–IV/I–II)	1.48 (0.81–3.66)	0.190		
Comorbidity: yes/no	2.33 (1.15–5.20)	0.045	1.54 (0.73–9.21)	0.510
Previous abdominal history: yes/no	0.92 (0.88–2.91)	0.780		
Tumor location				
Rectum	Reference	–		
Left colon	1.42 (0.71–7.21)	0.641		
Right colon	2.34 (0.90–6.22)	0.200		
Clinical TNM stage: III/II	3.25 (1.29–8.44)	0.017	2.42 (0.93–8.44)	0.159
Differentiation				
Well	Reference	–		
Moderate	1.20 (0.71–7.32)	0.492		
Poor	1.54 (0.89–5.44)	0.155		
Radical surgery (no/yes)	5.33 (2.13–18.32)	<0.001	4.32 (1.93–12.45)	<0.001
Postoperative complication (yes/no)	1.34 (0.74–2.40)	0.335		
Grade 3–4 postoperative complication (yes/no)	1.71 (0.84–4.94)	0.102		

## 4. Discussion

With the increasingly severe aging of China's population, the proportion of elderly individuals with colorectal cancer is increasing (4–6). Because elderly patients are insensitive to pain and slow to respond, disease progression is more insidious and often in the advanced stages at the time of presentation. Intestinal obstruction is the main complaint of elderly patients presenting with colorectal cancer. Elderly patients often have more underlying diseases and poor organ reserve, resulting in high surgical risks (8, 13, 14). Meanwhile, the choice of reasonable treatment and surgical method directly affects the perioperative safety and prognosis of elderly patients with colorectal cancer (9–11). Therefore, the present study aimed to compare the safety and long-term prognosis of palliative surgery and radical surgery in elderly colorectal cancer patients over 80 years old with intestinal obstruction.

Palliative surgery is often performed in patients who cannot achieve R0 resection or those with poor general condition and a high risk of surgical anesthesia. It is not objective and accurate to compare the long-term survival of patients undergoing radical surgery and palliative surgery. Therefore, this study excluded patients with distant metastasis and the inability to achieve R0 resection. In addition, patients with an ASA score of 4 or above or an ECOG score of 2 or above were also excluded. PSM was adopted to further eliminate the interference caused by confounding factors between the two groups. Therefore, we believe that the implementation of this study is relatively scientific and objective. The results of this study showed that the incidence of grade 1–5 complications (37.9% vs. 15.5%, *P *= 0.006) and gastrointestinal disorders (20.7% vs. 6.9%, *P *= 0.031) in the radical group were significantly higher than those in the palliative group. There was no significant difference in the incidence of grade 3–5 complications (6.9% vs. 1.7%, *P *= 0.364) between the two groups, and no perioperative deaths occurred. Elderly patients have poor functional reserves of the heart, lungs and other organs, and when intestinal obstruction occurs, anemia, hypoproteinaemia, electrolyte imbalance and other triggers will further reduce surgical tolerance. In addition, intestinal wall edema and intestinal flora disorders caused by intestinal obstruction will further increase the occurrence of gastrointestinal disorders such as anastomotic leakage and ileus. However, radical surgery is safe and feasible without increasing the incidence of serious postoperative complications and mortality in elderly patients with intestinal obstruction through adequate preoperative assessment, curated protection during surgery, and close postoperative monitoring.

Relief of obstruction is the primary goal of treatment for elderly colorectal cancer patients with intestinal obstruction. Considering objective factors such as the patient's general condition, surgical risk and family members' wishes, surgeons often adopt conservative treatment, such as ostomy, intestinal short-circuiting, or intestinal stent placement, but abandon radical surgery. With the continuous improvement of medical technology, the life expectancy of the elderly has been considerably extended. Under the premise of controllable surgical risks, elderly patients with colorectal cancer over 80 years old can also achieve important survival benefits through radical surgery. Since elderly patients often die due to various causes, this study evaluated the survival benefits provided by radical and palliative surgery in elderly patients by measuring the 5-year OS. The results of this study showed that patients who underwent radical surgery had a significantly better 5-year OS than those who underwent palliative surgery (55.2% vs. 22.6%, *P *< 0.001). In addition, various factors that might influence prognosis were included in a multivariate Cox analysis, and the results showed that palliative surgery (HR: 4.32; 95% CI, 1.93–12.45; *P *< 0.001) was an independent factor affecting poor prognosis in elderly colorectal cancer patients with intestinal obstruction. A study conducted by Takeuchi et al. classified 114 elderly colorectal cancer patients into two groups by age, and the results showed that the incidence of perioperative pulmonary complications (*P *= 0.0019) and mortality (*P *= 0.0447) in patients aged ≥85 years were significantly higher than those in patients aged <85 years. However, there was no significant difference in 2-year and 5-year OS between the two groups (15). Moreover, Bruce et al. proposed that advanced age should not be used as a contraindication to radical surgery, and radical resection of primary cancer and metastases can also be performed for elderly colorectal cancer patients with liver metastasis. Even if metastases cannot be resected at the same time, the treatment effect of only resection of the primary tumor is better than that of ostomy or intestinal short-circuiting (16). Therefore, we suggest that with the extension of life expectancy and the improvement of medical equipment technology, for elderly patients over 80 years old with intestinal obstruction, under the premise that the risk of anesthesia is controllable and curative resection is available, the opportunity for radical surgery should not be denied just because of advanced age.

With the extension of life expectancy and the continuous improvement of surgical anesthesia technology, the contraindication of radical surgery is often no longer advanced age. In the future, it is necessary to distinguish between the concepts of chronological age and frailty. Elderly colorectal cancer patients with good systemic condition can often benefit from radical surgery, while for frail patients with poor systemic nutrition, the significance and value of radical surgery should be fully considered as appropriate (17, 18).

The present study has some limitations that need to be declared. First, patients undergoing palliative surgery tend to have a poor general condition and advanced tumor stage, which can lead to a poor prognosis. Second, the study period was from 2012 to 2021, and the treatment strategies adopted by the included patients were inconsistent. In addition, confounding factors such as date of surgery and surgical approach will also indirectly affect the analysis of results. However, we used propensity score matching to reduce the above selection bias. Finally, the retrospective nature and small sample size of only 187 patients included were also limitations of this study.

## 5. Conclusion

Although elderly colorectal cancer patients over 80 years of age with intestinal obstruction are more likely to be admitted to the ICU and develop more postoperative complications after radical surgery, long-term survival benefits can be achieved. With the extension of life expectancy and the improvement of medical equipment technology, the opportunity for radical surgery should not be denied just because of advanced age.

## Data Availability

The raw data supporting the conclusions of this article will be made available by the authors, without undue reservation.
